# Pre- and post-diagnosis physical activity, television viewing, and mortality among hematologic cancer survivors

**DOI:** 10.1371/journal.pone.0192078

**Published:** 2018-01-31

**Authors:** Daniela Schmid, Gundula Behrens, Hannah Arem, Christina Hart, Wolfgang Herr, Carmen Jochem, Charles E. Matthews, Michael F. Leitzmann

**Affiliations:** 1 Department of Epidemiology and Preventive Medicine University of Regensburg, Regensburg, Germany; 2 Department of Epidemiology and Biostatistics, Milken Institute School of Public Health, George Washington University, Washington, D.C., United States of America; 3 Department of Internal Medicine III, Haematology & Oncology, University Hospital of Regensburg, Regensburg, Germany; 4 Division of Cancer Epidemiology and Genetics, Metabolic Epidemiology Branch, National Cancer Institute, Bethesda, MD, United States of America; University of Rochester, UNITED STATES

## Abstract

**Purpose:**

The associations of physical activity and television (TV) viewing with mortality risk among individuals with hematologic malignancies remain unclear.

**Methods:**

We examined the relations of physical activity and TV viewing time before and after diagnosis with mortality among 5182 U.S. adults aged 50–71 years from the NIH-AARP Diet and Health Study cohort who survived a first primary hematologic cancer between 1995–1996 and 2011.

**Results:**

For the pre- and post-diagnosis analyses, we confirmed 2606 and 613 deaths respectively. In multivariable-adjusted Cox proportional hazard regression models, comparing high (≥4 hrs/wk) versus low (<1 hr/wk) activity levels, pre-diagnosis physical activity was associated with 18%-22% reduced risks of all-cause mortality among all hematologic cancer survivors, and survivors of non-Hodgkin lymphoma, myeloma, and leukemia, respectively. Additional control for BMI had little impact on the results, expect for myeloma survivors, for whom the association was no longer significant. Post-diagnosis physical activity was related to risk reductions in mortality ranging from 36%-47%. The associations for TV viewing did not show a clear pattern.

**Conclusion:**

Our study suggests that pre- and post-diagnosis physical activity is associated with lower risk of all-cause mortality among hematologic cancer survivors. Further research is required to confirm this observation.

## Introduction

Hematologic cancers represent a group of malignant neoplasms of the hematopoietic and lymphoid tissues comprising Hodgkin lymphoma (HL), non-Hodgkin lymphoma (NHL), multiple myeloma, and leukemia. The survival rates for hematologic cancer have increased notably during the past few decades [1], seemingly as a result of improved diagnosis and health care services, and population aging. In the USA, 5-year survival rates of HL, NHL, and leukemia have reached 88%, 71% and 59%, respectively [1].

Cancer patients frequently experience fatigue, depression, and decreased physical function and quality of life, which are partly the consequences of cancer treatment [2–7]. Moreover, they are at increased risk of cancer recurrence, second malignancies, other chronic diseases, and premature death [8–10]. Exercise plays numerous roles to improve health among individuals presenting with cancer [11, 12], yet it remains unclear whether physical activity improves survival among hematologic cancer patients.

Two randomized controlled studies investigated physical activity in relation to mortality among 122 lymphoma patients [13] and 103 allogeneic stem cell transplant patients [14], one of which reported improved survival in the exercise group compared with controls [14], whereas the other failed to show a statistically significant relation of physical activity to progression-free survival [13]. A recent study [15] prospectively followed 238 cases of diffuse large B-cell lymphoma (DLBCL) and 175 cases of follicular lymphoma and found a 44% reduction in mortality among DLBCL survivors who were highly physically active before diagnosis compared with those with low physical activity. However, these studies [13–15] were potentially limited due to small sample size.

In addition to physical activity, television (TV) viewing time, one of the most prevalent sedentary behaviors, may affect cancer outcomes. The few available observational studies on TV viewing/leisure sitting time and cancer survival reported conflicting results and were restricted to cancers of the colorectum [16–18], breast [19], and endometrium [20].

We assembled data from the NIH-AARP Diet and Health Study cohort to examine whether moderate to vigorous intensity physical activity (MVPA) and TV viewing are related to all-cause and cancer-specific mortality risk among individuals diagnosed with hematologic cancer, including HL, NHL, multiple myeloma, and leukemia. We further explored the combined effects of MVPA and TV viewing time on mortality risk.

## Methods

### Study population

The NIH-AARP Diet and Health Study is a prospective cohort study of 566,398 adults aged 50–71 years, who completed a mailed baseline questionnaire in 1995–1996 [21]. Individuals without a history of cancer at baseline who were diagnosed with hematologic cancer between study entry and 2011 were eligible for this study. Demographic characteristics and medical history were used from the baseline questionnaire. For the pre-diagnosis analysis, physical activity and TV viewing were collected from the risk factor questionnaire, which was mailed within six months to those without colon, breast or prostate cancer at baseline (about 60% of the original cohort) and inquired detailed information about MVPA and time spent watching TV. The follow-up questionnaire sent to participants in 2004–2005 was used to define MVPA and TV viewing for the post-diagnosis analysis. We excluded participants with missing data on physical activity or TV viewing in the risk factor questionnaire (pre-diagnosis analysis) or follow-up questionnaire (post-diagnosis analysis), those with missing data on body mass index (BMI, calculated as weight in kilograms divided by height in meters squared) at baseline, those with a BMI <18.5 kg/m^2^ or >65 kg/m^2^ at baseline, those who died before exposure assessment, and those who were diagnosed at the date of/after the follow-up questionnaire entry.

The final analytic cohort comprised 5135 hematologic cancer survivors (2585 deaths) for the pre-diagnosis physical activity and 5182 (2606 deaths) for TV viewing analyses. A total of 1773 individuals (613 deaths) and 1636 individuals (577 deaths) were included to examine the relations of post-diagnosis physical activity and TV viewing with mortality outcomes. The NIH–AARP Diet and Health study was approved by the Special Studies Institutional Review Board of the US National Cancer Institute.

### Assessment of physical activity and TV viewing

In the risk factor questionnaire, study members were asked to report how often they participated in leisure time MVPA in the past 10 years: never, rarely, <1, 1 to 3, 4 to 7, or >7 h/wk. A list of examples of MVPA was provided including tennis, golf (walking), biking, swimming, heavy gardening, weight lifting, basketball/baseball, football/soccer, cheerleading/drill team, handball/racquetball, hiking/climbing mountains, fast walking/fast dancing, rowing, aerobics, jogging/running, and heavy housework. This questionnaire also inquired about hours watching TV or videos per day over the past 12 months: <1, 1 to 2, 3 to 4, 5 to 6, 7 to 8, or ≥9 h/d. We collapsed the categories for each exposure into three categories (MVPA: <1, 1 to 3, and ≥4 h/wk; TV viewing: 0 to 2, 3 to 4, and ≥5 h/d). In the follow-up questionnaire, participants were asked to report the weekly time they spent doing MVPA over the prior 12 months, choosing from eight exercise and recreational activities and 10 duration categories ranging from none to more than 10 h/week for each activity. Participants also reported the hours per day they spent sitting watching TV, video, or DVD in the previous 12 months, choosing from eight response categories ranging from 0 to >12 h/d. We created categories for physical activity and TV viewing that are comparable to those of the pre-diagnosis analysis. The risk factor questionnaire has not been internally validated, but the questions are similar to assessments of recreational MVPA and TV viewing reported to have reasonable validity [22–25]. The follow-up questionnaire items showed reasonable correlations with objective measurements of physical activity and sitting time [26].

### Ascertainment of hematologic cancer incidence and mortality

We identified hematologic cancer cases through the end of 2011 using linkage to eleven state cancer registry databases. Hematologic cancer cases, including HL, NHL, myeloma, leukemia were classified according to the histology codes from the International Classification of Diseases for Oncology, 3rd Edition (ICD-O-3) [27]. Leukemia comprised lymphocytic leukemia, myeloid and monocytic leukemia, and other leukemia. Vital status was identified by annual linkage of the cohort to the Social Security Administration Death Master File [28] and verified by the National Death Index (NDI) Plus [29]. We used ICD-9 and ICD-10 codes to classify mortality from hematologic cancer.

### Statistical analysis

Cox proportional hazard regression models were used to estimate hazard ratios (HRs) and 95% confidence intervals (CI) for mortality across different levels of MVPA, TV viewing time, and the combination of both exposures. We tested for the proportional hazards assumption using Schoenfeld residuals [30]. For the pre-diagnosis analysis, follow-up was calculated from the date of cancer diagnosis until either the date of death or December 31, 2011. In the post-diagnosis model, follow-up began on the date of follow-up questionnaire entry.

The basic model included age at exposure assessment, age at diagnosis, and sex. In a subsequent model, we entered education, race, smoking, alcohol consumption, chemotherapy (first course of treatment), hematologic cancer subtype, and stage in NHL survivors, and mutually adjusted for MVPA and TV viewing. The full model additionally included BMI. In supplementary analyses, we additionally adjusted for self-reported health status (baseline questionnaire for pre-diagnosis analysis and follow-up questionnaire for post-diagnosis analysis).

For trend analyses, we fit a continuous variable assigning each exposure the midpoint of its respective category. To account for the potential of reverse causation, which could have occurred if cancer patients had been less physically active due to symptoms of the disease at the time of physical activity assessment, we performed sensitivity analyses excluding those who answered the questionnaire within a year of diagnosis. In addition, we excluded individuals who died within a year of answering pre- and post-diagnosis questionnaires.

We further investigated whether the association of MVPA and TV viewing time with all-cause mortality was modified by age at diagnosis, sex, BMI, lag time between exposure assessment and cancer diagnosis, and pre-diagnosis exposures (post-diagnosis analysis). We performed interaction tests using likelihood ratio tests. All analyses were conducted using SAS version 9.4 (SAS Institute Inc., Cary, NC). P values were considered statistically significant at the 5% level.

## Results

The average times from cancer diagnosis to the end of follow-up and from follow-up questionnaire entry to the end of follow-up were 4.4 and 5.7 years, respectively. Study characteristics of the included participants are presented in Table 1. Individuals with a low MVPA level and those with a high level of TV viewing time were more likely to currently smoke than physically active individuals and those who spent less time watching TV. The physically active survivors were also more likely to report excellent or very good health. There were no important differences in other study characteristics among the study members.

**Table 1:** Baseline characteristics of the study participants diagnosed with incident hematologic cancer according to pre-diagnosis physical activity and TV viewing.

Characteristics		Pre-diagnosis physical activity			Pre-diagnosis TV viewing		
		<1 hr/wk	1 to 3 hrs/wk	≥4 hrs/week	0 to 2 hrs/d	3 to 4 hrs/d	≥5 hrs/d
Number of participants		1278	1272	2585	1750	2394	1038
Hematologic cancer subtype, %							
Hodgkin lymphoma		1.8	1.9	2.4	2.0	2.0	2.6
Non-Hodgkin lymphoma		49.4	52.9	49.7	53.5	49.4	47.4
Myeloma		16.3	15.7	16.8	16.0	16.3	17.0
Leukemia		32.4	29.5	31.1	28.6	32.2	33.0
Age at exposure assessment, years, mean (SD)		63.5 (5.1)	63.7 (5.0)	64.3 (4.8)	63.3 (5.2)	64.1 (4.8)	64.6 (4.7)
Age at diagnosis, years, mean (SD)		71.0 (6.4)	71.3 (6.4)	72.0 (6.1)	71.1 (6.5)	71.8 (6.2)	71.9 (6.1)
Sex, %							
Men		66.9	68.2	69.0	68.6	68.5	66.1
Women		33.1	31.8	31.0	31.4	31.5	33.9
Non-Hispanic White, %		92.8	94.3	94.4	95.2	94.4	90.9
Pre-diagnosis smoking status, %							
Never smoker		34.7	35.0	36.7	42.9	32.0	32.2
Past smoker		49.5	52.9	52.5	47.5	54.9	51.9
Current smoker		11.8	9.7	7.8	6.7	10.0	12.3
College graduate/post graduate, %		41.0	43.2	44.3	56.7	39.6	27.4
Pre-diagnosis body mass index, kg/m^2^, mean (SD)		28.6 (5.3)	27.5 (4.6)	26.6 (3.9)	26.4 (4.0)	27.5 (4.5)	28.6 (5.2)
Pre-diagnosis alcohol intake, %							
0 g/d		28.0	21.8	21.4	23.1	22.0	26.0
>0 to 14.9 g/d		54.0	57.7	55.4	55.3	55.5	56.5
≥15 g/d		18.0	20.6	23.1	21.6	22.5	17.5
Chemotherapy (first course of treatment), %		28.6	26.7	29.0	29.3	28.0	27.9
Pre-diagnosis self-reported heath status, %							
Excellent/very good		40.5	49.6	62.2	62.9	52.5	39.8
Good		40.2	39.9	30.0	29.5	36.1	40.9
Fair/poor		17.3	8.9	6.6	6.3	9.8	17.7

All values (except age) are standardized to the age distribution of the study population. Categories may not sum up to 100% due to missing data. Smoking status, body mass index, alcohol intake, and self-reported health status were assessed in the baseline questionnaire.

### Physical activity

Compared with engaging in <1 h/wk of MVPA, ≥4 h/wk of MVPA before diagnosis showed 18% (multivariable-adjusted HR = 0.82, 95% CI = 0.74–0.90), 18% (HR = 0.82, 95% CI = 0.71–0.95), and 19% (HR = 0.81, 95% CI = 0.69–0.95) reduced risks of all-cause mortality among all hematologic survivors, NHL survivors, and leukemia survivors, respectively, (Table 2). Among leukemia survivors, we found a stronger inverse association with pre-diagnosis physical activity for chronic leukemia (HR = 0.65, 95% CI = 0.50–0.83) than acute leukemia (HR = 0.90, 95% CI = 0.72–1.13). We noted an inverse relation of physical activity before diagnosis to mortality risk among myeloma survivors (multivariable-adjusted HR = 0.78, 95% CI = 0.63–0.96), but that association was no longer statistically significant after additional control for BMI (HR = 0.81, 95% CI = 0.66–1.01). Among hematologic cancer survivors, we noted an inverse association of pre- and post-diagnosis MVPA with mortality from hematologic cancer, although the results for post-diagnosis MVPA did not reach statistical significance in the multivariable-adjusted model (Table 2).

**Table 2:** HRs and 95% CI of mortality among individuals diagnosed with hematologic cancer according to physical activity before and after diagnosis.

	Pre-diagnosis physical activity				Post-diagnosis physical activity			
	<1 hr/wk	1 to 3 hrs/wk	≥4 hrs/wk	*p*-trend	<1 hr/wk	1 to <4 hrs/wk	≥4 hrs/wk	*p*-trend
**All hematologic cancer survivors**								
**All-cause mortality**								
Person-years	5121	5749	11637		2460	3595	4001	
Deaths	701	623	1261		206	216	191	
Model 1	1.00	0.81 (0.73–0.90)	0.78 (0.71–0.86)	<0.001	1.00	0.72 (0.59–0.87)	0.56 (0.46–0.68)	<0.001
Model 2	1.00	0.85 (0.76–0.95)	0.82 (0.74–0.90)	<0.001	1.00	0.76 (0.63–0.93)	0.61 (0.50–0.74)	<0.001
Model 3	1.00	0.87 (0.78–0.97)	0.85 (0.77–0.93)	0.005	1.00	0.77 (0.63–0.94)	0.61 (0.50–0.75)	<0.001
**Hematologic cancer mortality**								
Deaths	485	407	891		95	122	110	
Model 1	1.00	0.77 (0.68–0.88)	0.82 (0.73–0.91)	0.02	1.00	0.87 (0.67–1.14)	0.69 (0.52–0.91)	0.01
Model 2	1.00	0.81 (0.71–0.92)	0.84 (0.75–0.94)	0.03	1.00	0.95 (0.72–1.25)	0.79 (0.59–1.05)	0.08
Model 3	1.00	0.83 (0.72–0.94)	0.87 (0.77–0.97)	0.12	1.00	0.95 (0.72–1.25)	0.79 (0.59–1.06)	0.09
**Non-Hodgkin lymphoma survivors**								
**All-cause mortality**								
Deaths	295	281	523		85	110	90	
Model 1	1.00	0.84 (0.72–0.99)	0.77 (0.67–0.89)	0.001	1.00	0.88 (0.66–1.17)	0.62 (0.46–0.84)	0.001
Model 2	1.00	0.89 (0.75–1.05)	0.82 (0.71–0.95)	0.01	1.00	0.91 (0.68–1.22)	0.63 (0.47–0.86)	0.002
Model 3	1.00	0.90 (0.77–1.07)	0.85 (0.73–0.99)	0.03	1.00	0.93 (0.69–1.24)	0.64 (0.47–0.87)	0.003
**Myeloma survivors**								
**All-cause mortality**								
Deaths	137	119	274		46	41	34	
Model 1	1.00	0.79 (0.62–1.01)	0.86 (0.70–1.05)	0.42	1.00	0.59 (0.39–0.91)	0.47 (0.30–0.75)	0.002
Model 2	1.00	0.75 (0.58–0.96)	0.78 (0.63–0.96)	0.10	1.00	0.65 (0.40–1.05)	0.52 (0.31–0.85)	0.01
Model 3	1.00	0.76 (0.59–0.98)	0.81 (0.66–1.01)	0.24	1.00	0.66 (0.41–1.06)	0.53 (0.32–0.88)	0.02
**Leukemia survivors**								
**All-cause mortality**								
Deaths	254	211	439		68	61	62	
Model 1	1.00	0.82 (0.69–0.99)	0.77 (0.66–0.90)	0.003	1.00	0.63 (0.44–0.89)	0.55 (0.39–0.78)	0.002
Model 2	1.00	0.88 (0.73–1.06)	0.81 (0.69–0.95)	0.01	1.00	0.65 (0.45–0.93)	0.58 (0.40–0.84)	0.01
Model 3	1.00	0.91 (0.75–1.09)	0.84 (0.72–0.99)	0.048	1.00	0.65 (0.45–0.94)	0.56 (0.38–0.83)	0.01
Acute leukemia survivors*								
**All-cause mortality**								
Deaths	134	97	235					
Model 1	1.00	0.81 (0.62–1.05)	0.83 (0.67–1.03)	0.20	-	-	-	-
Model 2	1.00	0.76 (0.58–1.01)	0.90 (0.72–1.13)	0.83	-	-	-	-
Model 3	1.00	0.79 (0.60–1.05)	0.94 (0.75–1.18)	0.94	-	-	-	-
**Chronic leukemia survivors**								
**All-cause mortality**								
Deaths	105	100	170		57	49	49	
Model 1	1.00	0.91 (0.69–1.20)	0.65 (0.51–0.83)	<0.001	1.00	0.66 (0.45–0.97)	0.54 (0.36–0.79)	0.003
Model 2	1.00	0.92 (0.69–1.22)	0.65 (0.50–0.83)	<0.001	1.00	0.70 (0.47–1.05)	0.57 (0.38–0.86)	0.01
Model 3	1.00	0.93 (0.70–1.24)	0.67 (0.51–0.87)	0.001	1.00	0.68 (0.45–1.03)	0.54 (0.35–0.83)	0.01

HR = hazard ratio, CI = confidence interval. Model 1: adjusted for age at exposure assessment (continuous), age at cancer diagnosis (continuous), and sex. Model 2: adjusted for all variables in Model 1 and additionally adjusted for education (less than 12 yrs, 12 yrs, vocational training or some college education, college graduate/postgraduate, unknown), race (non-Hispanic White, non-Hispanic Black, other, unknown), smoking (never smoker, former smoker with 20 cigarettes per day or less, former smoker with more than 20 cigarettes per day, current smoker with 20 cigarettes per day or less, current smoker with more than 20 cigarettes per day, missing), alcohol consumption (0, >0 to 14.9, ≥15g/d), chemotherapy (yes, no, unknown/missing), hematologic cancer subtype (NHL, HL, myeloma, leukemia) and stage in NHL survivors (localized/regional/in situ, systemic disease, unknown/not abstracted/missing), and TV viewing. Model 3: adjusted for all variables in Model 2 and additionally adjusted for body mass index (18.5-<25.0kg/m^2^, 25.0-<30.0 kg/m^2^, 30.0-<35.kg/m^2^, 35-<65 kg/m^2^).

* Data were not evaluated for post-diagnosis physical activity due to low sample size (n = 23).

Physical activity performed after diagnosis yielded multivariable-adjusted HRs of all-cause mortality of 0.61 (95% CI = 0.50–0.74), 0.63 (95% CI = 0.47–0.86), 0.52 (95% CI = 0.31–0.85), and 0.58 (95% CI = 0.40–0.84) among all hematologic cancer survivors, NHL survivors, myeloma survivors, and leukemia survivors, respectively (Table 2). In a sub-analysis, we restricted the study population to individuals who provided information on both pre- and post-diagnosis exposures and noted that the inverse association of post-diagnosis MVPA with mortality among NHL survivors was attenuated following additional control for pre-diagnosis MVPA (S1 Table).

Due to low sample size (n = 52), we were not able to explore relations among HL survivors.

### TV viewing

Hematologic cancer survivors watching TV ≥5 hours daily before diagnosis had a 14% increased risk of all-cause mortality risk compared with those spending 0 to 2 hrs/d watching TV (multivariable-adjusted HR = 1.14, 95% CI = 1.02–1.28), but the association was no longer statistically significant following additional control for BMI (HR = 1.10, 95% CI = 0.99–1.24, Table 3). We noted a statistically significant positive association between pre-diagnosis TV viewing and mortality among survivors of NHL and acute leukemia.

**Table 3:** HRs and 95% CI of mortality among individuals diagnosed with hematologic cancer according to TV viewing before and after diagnosis.

	Pre-diagnosis TV viewing				Post-diagnosis TV viewing			
	0 to 2 hrs/d	3 to 4 hrs/d	≥5 hrs/d	*p*-trend	0 to 2 hrs/d	>2 to 4 hrs/d	>4 hrs/d	*p*-trend
**All-cause mortality**								
Person-years	8127	10223	4426		3560	3439	2240	
Deaths	774	1239	593		201	199	177	
Model 1	1.00	1.20 (1.10–1.32)	1.30 (1.17–1.45)	<0.001	1.00	1.00 (0.82–1.22)	1.41 (1.15–1.72)	0.003
Model 2	1.00	1.11 (1.01–1.21)	1.14 (1.02–1.28)	0.01	1.00	0.94 (0.77–1.14)	1.20 (0.97–1.48)	0.15
Model 3	1.00	1.09 (0.99–1.19)	1.10 (0.99–1.24)	0.06	1.00	0.93 (0.77–1.14)	1.19 (0.96–1.47)	0.19
**Hematologic cancer mortality**								
Deaths	553	851	391		109	111	86	
Model 1	1.00	1.16 (1.04–1.29)	1.21 (1.07–1.38)	0.002	1.00	1.05 (0.80–1.36)	1.29 (0.97–1.72)	0.10
Model 2	1.00	1.08 (0.97–1.21)	1.10 (0.96–1.25)	0.14	1.00	0.99 (0.76–1.30)	1.13 (0.84–1.52)	0.47
Model 3	1.00	1.07 (0.96–1.19)	1.06 (0.93–1.22)	0.32	1.00	0.99 (0.76–1.29)	1.12 (0.83–1.52)	0.51
**Non-Hodgkin lymphoma survivors**								
**All-cause mortality**								
Deaths	337	524	247		100	96	76	
Model 1	1.00	1.27 (1.10–1.45)	1.39 (1.18–1.64)	<0.001	1.00	1.03 (0.78–1.37)	1.30 (0.96–1.75)	0.12
Model 2	1.00	1.18 (1.02–1.36)	1.25 (1.05–1.49)	0.01	1.00	1.04 (0.78–1.38)	1.17 (0.86–1.60)	0.34
Model 3	1.00	1.16 (1.01–1.34)	1.23 (1.03–1.46)	0.01	1.00	1.03 (0.78–1.38)	1.15 (0.84–1.58)	0.41
**Myeloma survivors**								
**All-cause mortality**								
Deaths	173	244	119		41	36	33	
Model 1	1.00	0.90 (0.74–1.10)	0.86 (0.68–1.09)	0.19	1.00	0.87 (0.55–1.36)	1.08 (0.68–1.73)	0.84
Model 2	1.00	0.85 (0.70–1.05)	0.79 (0.61–1.02)	0.05	1.00	0.86 (0.52–1.43)	0.98 (0.57–1.67)	0.89
Model 3	1.00	0.85 (0.69–1.04)	0.76 (0.59–0.98)	0.03	1.00	0.85 (0.51–1.42)	0.92 (0.53–1.59)	0.73
**Leukemia survivors**								
**All-cause mortality**								
Deaths	253	446	211		55	61	63	
Model 1	1.00	1.19 (1.02–1.39)	1.28 (1.07–1.54)	0.01	1.00	0.99 (0.69–1.43)	1.65 (1.15–2.37)	0.01
Model 2	1.00	1.17 (0.996–1.37)	1.19 (0.99–1.45)	0.05	1.00	0.86 (0.59–1.25)	1.46 (0.99–2.16)	0.09
Model 3	1.00	1.15 (0.98–1.35)	1.15 (0.95–1.40)	0.11	1.00	0.86 (0.59–1.25)	1.50 (1.003–2.25)	0.09
Acute leukemia survivors*								
**All-cause mortality**								
Deaths	135	224	111					
Model 1	1.00	1.29 (1.04–1.61)	1.51 (1.18–1.95)	0.001		-	-	-
Model 2	1.00	1.29 (1.03–1.60)	1.46 (1.13–1.91)	0.003		-	-	-
Model 3	1.00	1.27 (1.01–1.58)	1.39 (1.06–1.82)	0.01		-	-	-
**Chronic leukemia survivors**								
**All-cause mortality**								
Deaths	104	190	82		42	49	54	
Model 1	1.00	1.16 (0.91–1.48)	1.08 (0.81–1.44)	0.50	1.00	0.98 (0.65–1.48)	1.71 (1.14–2.56)	0.01
Model 2	1.00	1.16 (0.91–1.49)	0.99 (0.73–1.34)	0.89	1.00	0.84 (0.54–1.29)	1.57 (1.02–2.42)	0.06
Model 3	1.00	1.13 (0.88–1.46)	0.95 (0.69–1.29)	0.88	1.00	0.86 (0.55–1.32)	1.69 (1.07–2.67)	0.04

HR = hazard ratio, CI = confidence interval, TV = television, BMI = body mass index. Model 1: adjusted for age at exposure assessment (continuous), age at cancer diagnosis (continuous), and sex. Model 2: adjusted for all variables in Model 1 and additionally adjusted for education (less than 12 yrs, 12 yrs, vocational training or some college education, college graduate/postgraduate, unknown), race (non-Hispanic White, non-Hispanic Black, other, unknown), smoking (never smoker, former smoker with 20 cigarettes per day or less, former smoker with more than 20 cigarettes per day, current smoker with 20 cigarettes per day or less, current smoker with more than 20 cigarettes per day, missing), alcohol consumption (0, >0 to 14.9, ≥15g/d), chemotherapy (yes, no, unknown/missing), hematologic cancer subtype (NHL, HL, myeloma, leukemia) and stage in NHL survivors (localized/regional/in situ, systemic disease, unknown/not abstracted/missing), and physical activity. Model 3: adjusted for all variables in Model 2 and additionally adjusted for body mass index (18.5-<25.0kg/m^2^, 25.0-<30.0 kg/m^2^, 30.0-<35.kg/m^2^, 35-<65 kg/m^2^). Data were not evaluated for post-diagnosis physical activity due to low sample size (n = 23).

*Data were not evaluated for post-diagnosis TV viewing due to low sample size (n = 21)

High vs. low post-diagnosis TV viewing was related to an increased mortality risk among all leukemia survivors (HR = 1.50, 95% CI = 1.003–2.25) and chronic leukemia survivors (HR = 1.69, 95% CI = 1.07–2.67, Table 3) in the most comprehensively adjusted model. Among hematologic cancer survivors who provided information on both pre- and post-diagnosis exposures, we observed a statistically significant positive association between post-diagnosis TV viewing and mortality in models that were additionally adjusted for pre-diagnosis TV viewing (S1 Table).

### Joint associations of physical activity and TV viewing

In an evaluation of joint associations of MVPA and TV viewing with risk of all-cause mortality, we noted that the combination of regular pre-diagnosis MVPA (≥1 hr/wk) and low levels of pre-diagnosis TV viewing (0 to 2 hrs/d) was related to reduced mortality risk among all hematologic cancer survivors, NHL survivors, and leukemia survivors compared to low MVPA levels (<1 hr/wk) and a high volume of TV viewing (≥3 hrs/wk) (Table 4). Moreover, regular post-diagnosis MVPA was associated with reduced mortality risk at both low and high volumes of TV viewing among all hematologic cancer survivors, NHL survivors, and leukemia survivors.

**Table 4:** Joint associations of pre- and post-physical activity and TV viewing with all-cause mortality among survivors of hematologic malignancies.

		TV viewing												
Physical activity		Pre-diagnosis												
		All hematologic cancer survivors		NHL survivors		Myeloma survivors			Leukemia survivors		Acute leukemia survivors*		Chronic leukemia survivors	
		High (≥3 to hrs/d)	Low (0 to 2 hrs/d)	High (≥3 to hrs/d)	Low (0 to 2 hrs/d)	High (≥3 to hrs/d)	Low (≥3 to hrs/d)		High (≥3 to hrs/d)	Low (0 to 2 hrs/d)	High (≥3 to hrs/d)	Low (0 to 2 hrs/d)	High (≥3 to hrs/d)	Low (0 to 2 hrs/d)
**Low (<1 h/wk)**	Deaths	519	178	209	85	102	34		196	56	103	31	80	23
	Multivariable-adjusted HR (95% CI)	1.00	0.91 (0.77–1.08)	1.00	0.91 (0.71–1.18)	1.00	1.16 (0.78–1.74)		1.00	0.89 (0.66–1.21)	1.00	0.61 (0.41–0.93)	1.00	0.98 (0.61–1.58)
**High (≥1 h/wk)**	Deaths	1292	587	552	250	255	137		456	192	230	102	190	78
	Multivariable-adjusted HR (95% CI)	0.83 (0.75–0.92)	0.74 (0.65–0.83)	0.87 (0.74–1.02)	0.71 (0.59–0.86)	0.75 (0.59–0.95)	0.93 (0.71–1.21)		0.85 (0.71–1.01)	0.70 (0.57–0.86)	0.79 (0.61–1.01)	0.64 (0.48–0.85)	0.77 (0.58–1.004)	0.66 (0.47–0.92)
		**Post-diagnosis**												
**Low (<1 h/wk)**	Deaths	140	57	55	29	30	12	50		14			42	12
	Multivariable-adjusted HR (95% CI)	1.00	0.83 (0.61–1.13)	1.00	0.83 (0.53–1.31)	1.00	0.76 (0.37–1.59)	1.00		0.85 (0.46–1.60)	-	-	1.00	0.89 (0.45–1.75)
**High (≥1 h/wk)**	Deaths	234	142	115	70	39	29	74		40			61	30
	Multivariable-adjusted HR (95% CI)	0.62 (0.5–0.77)	0.63 (0.50–0.81)	0.69 (0.50–0.95)	0.62 (0.43–0.90)	0.50 (0.29–0.85)	0.67 (0.36–1.24)	0.60 (0.41–0.88)		0.57 (0.37–0.89)	-	-	0.66 (0.43–1.01)	0.62 (0.38–1.02)

HR = hazard ratio, CI = confidence interval, TV = television, BMI = body mass index. Low PA = low physical activity (<1 hr/wk), High PA = high physical activity (≥1 hr/wk), Low TV = low television viewing (<3 hrs/d), High TV = high television viewing (≥3 hrs/d). Multivariable models are adjusted for age at exposure assessment (continuous), age at cancer diagnosis (continuous), education (less than 12 yrs, 12 yrs, vocational training or some college education, college graduate/postgraduate, unknown), race (non-Hispanic White, non-Hispanic Black, other, unknown), smoking (never smoker, former smoker with 20 cigarettes per day or less, former smoker with more than 20 cigarettes per day, current smoker with 20 cigarettes per day or less, current smoker with more than 20 cigarettes per day, missing), alcohol consumption (0, >0 to 14.9, ≥15g/d), hematologic cancer subtype (NHL, HL, myeloma, leukemia) and stage in NHL survivors (localized/regional/in situ, systemic disease, unknown/not abstracted/missing), and chemotherapy (yes, no, unknown/missing).

*Data were not evaluated for the post-diagnosis combination of physical activity and TV viewing due to low sample size.

### Sub-analyses

Stratified analysis indicated that post-diagnosis TV viewing was significantly related to increased mortality among all hematologic cancer survivors aged 69 years (median) and older at diagnosis (multivariable-adjusted HR = 1.51, 95% CI = 1.15–2.00), whereas a statistically non-significant inverse association was detected among those aged <69 years (HR = 0.89, 95% CI = 0.64–1.25, P-interaction = 0.03, S2 Table). Stratification by pre-diagnosis TV viewing time indicated a stronger positive association between high versus low levels of post-diagnosis TV viewing and mortality among those with a high amount of pre-diagnosis TV viewing time than those with lower amounts of pre-diagnosis TV viewing time, although the p for interaction was not statistically significant (p = 0.20, S3 Table).

In supplementary analyses, we additionally adjusted for self-reported health (S4 Table and S5 Table) and found that the MVPA and mortality relations among NHL and leukemia survivors were attenuated (S4 Table).

Excluding participants diagnosed with hematologic cancer within one year of exposure assessment or excluding participants who answered the questionnaire in the 12 months prior to death did not meaningfully alter the interpretation of the results on MVPA or TV viewing and all-cause mortality among all hematologic cancer survivors (S6 Table and S7 Table). Notably, the inverse associations of post-diagnosis MVPA and all-cause mortality among all leukemia survivors and chronic leukemia survivors were attenuated after excluding individuals who died within the first year of exposure assessment (S6 Table).

## Discussion

In this large prospective cohort study, increased MVPA both before and after diagnosis was associated with reduced risk of all-cause mortality among adult survivors of NHL, myeloma, and leukemia. Pre-diagnosis MVPA was also related to decreased risk of hematologic cancer-specific mortality. By comparison, the association between post-diagnosis MVPA and hematologic cancer-specific mortality was not statistically significant. The results for TV viewing and mortality risk did not show a clear pattern.

Data regarding the relationship between exercise and survival among hematologic cancer patients are limited to two randomized controlled trials and one prospective epidemiologic investigation [13–15]. The Healthy Exercise for Lymphoma Patients (HELP) Trial reported that random assignment to 12 weeks of exercise had no effect on survival among 122 lymphoma patients during the 61-month follow-up period. However, secondary analyses that broke the randomization and accounted for a potential cross-over effect of exercise among controls showed a statistically non-significant improvement in progression-free survival (HR = 0.69, 95% CI = 0.45–1.06) [13]. The other trial [14] found a significantly reduced mortality risk among 103 allogeneic stem cell transplant patients with a combination of endurance and resistance exercise (total mortality rates for exercise vs. control group: 12.0 vs. 28.3%, P = 0.03) over two years. However, those trials [13, 14] were not primarily designed to evaluate survival outcomes and were compromised by small sample sizes and heterogeneous patient populations. In a recent epidemiologic investigation among 238 DLBCL and 175 follicular lymphoma cases [15], a 41% risk reduction in mortality was found among DLBCL survivors comparing high versus low levels of pre-diagnosis physical activity.

Biologic mechanisms explaining the apparent protective effect of physical activity on mortality among hematologic cancer survivors are speculative. Physical activity may mediate numerous pathways associated with cancer progression and the development of other chronic diseases including energy metabolism, insulin, insulin-like growth factor-1, chronic inflammation, the immune system, and antioxidant pathways [31, 32]. We found that the pre-diagnosis MVPA and mortality relation among myeloma survivors was no longer statistically significant following additional control for BMI. Myeloma is more strongly related to BMI than other hematologic malignancies [33], stressing a potential mediator role of BMI for the MVPA and myeloma survival relation.

It has been suggested that cancer patients who exercise regularly before diagnosis are more likely to remain physically active post-diagnosis [34] and may be pre-disposed to biologically less aggressive tumors [16] showing a lower rate of relapse. Physical activity may also beneficially influence the treatment process [13, 35] through improved completion rates and treatment response, and the probability of receiving second- and third-line cancer treatments. Physical activity performed after diagnosis may reduce comorbidities [36], thereby lowering mortality risk.

In sensitivity analyses, the inverse association between post-diagnosis MVPA and all-cause mortality among leukemia survivors was attenuated after excluding deaths within 12 months of the activity assessment. We recognize that sick patients may have been less physically active after diagnosis due to symptoms and occult disease leading to reverse causation, which may have resulted in the strong inverse association noted for post-diagnosis activity.

Our study showed mixed results regarding TV viewing and survival, which may be due to chance, low sample size, or cancer-specific effects. We also found that the post-diagnosis TV viewing relation became statistically significant after additional control for pre-diagnosis TV viewing time suggesting that the relation for post-diagnosis TV viewing may be partly confounded by pre-diagnosis TV viewing. Moreover, the pre-diagnosis TV viewing and mortality association was attenuated after including BMI in the models. Obesity may represent a plausible biologic pathway by which sedentary behavior contributes to the development of chronic disease [37]. In animal studies, hindlimb unloading has been shown to suppress skeletal muscle lipoprotein lipase production, a key enzyme in the regulation of the glucose and lipid metabolism [38–40]. Other potential pathways include chronic inflammation, oxidative stress, sex hormones, and alterations in the immune system [37, 41]. A potential adverse effect may also be partly attributable to unhealthy eating [42] and smoking behaviors [43] associated with prolonged TV viewing.

Strengths of our study include its prospective design, adjustment for a number of important confounding variables, and availability of information on physical activity and TV viewing from two questionnaires which allowed conducting pre- and post-diagnosis analyses. Nevertheless, several limitations must be considered in terms of interpreting our findings. Our results by hematologic cancer subtype may have been underpowered by virtue of the low sample sizes in those subtype-specific analyses. As mentioned above, we cannot exclude the possibility that reverse causation due to occult disease at the time of exposure assessment may have affected our results. Moreover, we cannot rule out the potential for measurement error resulting from self-reported assessments of physical activity and TV viewing. Although we carefully controlled for a wide range of potential confounding variables, residual confounding through covariates measured with error is possible.

Our findings suggest that it is advisable to counsel hematologic cancer survivors to engage in regular physical activity to improve survival after diagnosis. The physical activity guidelines set forth by the U.S. Department of Health and Human Services and the American Cancer Society [44] advocate individuals affected by cancer to comply with the physical activity recommendations for the general adult population and to engage in at least 150 minutes of moderate activity per week, with the understanding that exercise prescription may need to be adjusted to their abilities and conditions.

In conclusion, our findings suggest improved survival attributable to high MVPA levels before and after diagnosis among survivors of hematologic malignancies. Our study did not show a clear pattern for TV viewing. In future studies, objective quantification of physical activity is required to determine the proper volume and duration of physical activity needed to improve cancer survival. Future work is also needed to clarify whether the relations differ across histologic subtypes taking into account clinical parameters such as cancer stage, treatment, and time since diagnosis. Finally, whether TV viewing increases risk of mortality among hematologic cancer survivors requires further evaluation.

## Supporting information

S1 Table Association of post-diagnosis physical activity and TV viewing with all-cause mortality in a subgroup of hematologic cancer survivors that provided information on pre- and post-diagnosis exposures, additionally adjusted for pre-diagnosis exposure. (DOCX)

S2 Table Multivariable-adjusted HRs and 95% CI of all-cause mortality among all individuals diagnosed with hematologic cancer according to physical activity and TV viewing before and after diagnosis, stratified by age at diagnosis, sex, BMI, and lag time between questionnaire administration and age at diagnosis. (DOCX)

S3 Table Multivariable-adjusted HRs and 95% CI for the relation of post-diagnosis physical activity or TV viewing to all-cause mortality among all individuals diagnosed with hematologic cancer according to pre-diagnosis physical activity or TV viewing. (DOCX)

S4 Table Multivariable-adjusted HRs and 95% CI of all-cause mortality among individuals diagnosed with hematologic cancer according to physical activity before and after diagnosis, additionally adjusted for self-reported health. (DOCX)

S5 Table Multivariable-adjusted HRs and 95% CI of all-cause mortality among individuals diagnosed with hematologic cancer according to TV viewing before and after diagnosis, additionally adjusted for self-reported health status. (DOCX)

S6 Table Multivariable-adjusted HRs and 95% CI of mortality according to physical activity before and after diagnosis among hematologic cancer survivors after exclusion of participants who answered the questionnaire within 12 months of death or participants diagnosed with hematologic cancer within one year of exposure assessment. (DOCX)

S7 Table Multivariable-adjusted HRs and 95% CI of mortality according to TV viewing before and after diagnosis among hematologic cancer survivors after exclusion of participants who answered the questionnaire within 12 months of death or participants diagnosed with hematologic cancer within one year of exposure assessment. (DOCX)
