# Supplementary material for: Pre- and post-diagnosis physical activity, television viewing, and mortality among hematologic cancer survivors
Source: PLoS One. 2018 Jan 31;13(1):e0192078. doi: 10.1371/journal.pone.0192078 (PMC5791989; doi:10.1371/journal.pone.0192078)
Supplement: S1 Table — (DOCX) [file pone.0192078.s001.docx]

**S1 Table.** Association of post-diagnosis physical activity and TV viewing with all-cause mortality in a subgroup of hematologic cancer survivors that provided information on pre- and post-diagnosis exposures, additionally adjusted for pre-diagnosis exposure.

|  | **Post-diagnosis physical activity** | | | | **Post-diagnosis TV viewing** | | | |
| --- | --- | --- | --- | --- | --- | --- | --- | --- |
| **All hematologic cancer survivors** | **<1 hr/wk** | **1 to <4 hrs/wk** | **≥4 hrs/wk** | ***p*-trend** | **0 to 2 hrs/d** | **>2 to 4 hrs/d** | **>4 hrs/d** | ***p*-trend** |
| Deaths | 132 | 163 | 134 |  | 143 | 139 | 122 |  |
| Model 1 | 1.00 | 0.83 (0.66-1.04) | 0.60 (0.47-0.77) | <0.001 | 1.00 | 0.96 (0.76-1.21) | 1.38 (1.08-1.76) | 0.02 |
| Model 2 | 1.00 | 0.90 (0.71-1.13) | 0.66 (0.51-0.85) | 0.001 | 1.00 | 0.94 (0.73-1.21) | 1.37 (1.04-1.81) | 0.05 |
| Model 3 | 1.00 | 0.89 (0.71-1.13) | 0.65 (0.51-0.85) | 0.001 | 1.00 | 0.93 (0.73-1.20) | 1.37 (1.03-1.81) | 0.06 |
| **Non-Hodgkin lymphoma** |  |  |  |  |  |  |  |  |
| Deaths | 54 | 85 | 62 |  | 67 | 64 | 56 |  |
| Model 1 | 1.00 | 1.06 (0.76-1.49) | 0.70 (0.48-1.003) | 0.02 | 1.00 | 1.01 (0.72-1.43) | 1.38 (0.96-1.97) | 0.11 |
| Model 2 | 1.00 | 1.18 (0.83-1.67) | 0.71 (0.48-1.04) | 0.04 | 1.00 | 0.97 (0.67-1.42) | 1.43 (0.94-2.19) | 0.16 |
| Model 3 | 1.00 | 1.17 (0.82-1.66) | 0.69 (0.47-1.03) | 0.03 | 1.00 | 0.98 (0.67-1.43) | 1.46 (0.95-2.24) | 0.14 |
| **Myeloma** |  |  |  |  |  |  |  |  |
| Deaths | 32 | 29 | 26 |  | 33 | 25 | 23 |  |
| Model 1 | 1.00 | 0.56 (0.34-0.93) | 0.45 (0.27-0.77) | 0.01 | 1.00 | 0.82 (0.48-1.38) | 1.16 (0.67-2.02) | 0.76 |
| Model 2 | 1.00 | 0.70 (0.39-1.24) | 0.49 (0.26-0.91) | 0.03 | 1.00 | 0.97 (0.52-1.81) | 1.19 (0.63-2.24) | 0.62 |
| Model 3 | 1.00 | 0.71 (0.40-1.27) | 0.49 (0.26-0.93) | 0.03 | 1.00 | 1.01 (0.54-1.89) | 1.19 (0.62-2.28) | 0.63 |
| **Leukemia** |  |  |  |  |  |  |  |  |
| Deaths | 41 | 45 | 43 |  | 40 | 44 | 40 |  |
| Model 1 | 1.00 | 0.77 (0.50-1.18) | 0.58 (0.38-0.90) | 0.02 | 1.00 | 0.89 (0.58-1.37) | 1.42 (0.91-2.20) | 0.18 |
| Model 2 | 1.00 | 0.80 (0.50-1.26) | 0.62 (0.39-1.001) | 0.05 | 1.00 | 0.72 (0.44-1.18) | 1.27 (0.74-2.17) | 0.47 |
| Model 3 | 1.00 | 0.79 (0.49-1.25) | 0.59 (0.36-0.96) | 0.03 | 1.00 | 0.70 (0.42-1.15) | 1.32 (0.76-2.31) | 0.44 |
| **Chronic leukemia** |  |  |  |  |  |  |  |  |
| Deaths | 34 | 35 | 34 |  | 33 | 33 | 34 |  |
| Model 1 | 1.00 | 0.78 (0.49-1.26) | 0.54 (0.34-0.87) | 0.01 | 1.00 | 0.73 (0.45-1.18) | 1.34 (0.82-2.17) | 0.36 |
| Model 2 | 1.00 | 0.88 (0.53-1.46) | 0.64 (0.37-1.09) | 0.09 | 1.00 | 0.57 (0.33-1.01) | 1.34 (0.73-2.45) | 0.43 |
| Model 3 | 1.00 | 0.79 (0.47-1.34) | 0.56 (0.32-0.97) | 0.04 | 1.00 | 0.59 (0.33-1.04) | 1.47 (0.78-2.77) | 0.35 |

HR=hazard ratio, CI=confidence interval, BMI=body mass index

Model 1: adjusted for age at exposure assessment (continuous), age at cancer diagnosis (continuous), and sex.

Model 2: additionally adjusted for education (less than 12 yrs, 12 yrs, vocational training or some college education, college graduate/postgraduate, unknown), race (Non-Hispanic White, non-Hispanic Black, other, unknown), smoking (never smoker, former smoker with 20 cigarettes per day or less, former smoker with more than 20 cigarettes per day, current smoker with 20 cigarettes per day or less, current smoker with more than 20 cigarettes per day, missing), alcohol consumption (0, >0 to 14.9, ≥15g/d), chemotherapy (yes, no, unknown/missing), hematologic cancer subtype (NHL, HL, myeloma, leukemia) and stage in NHL survivors (localized/regional/in situ, systemic disease, unknown/not abstracted/missing), post-diagnosis physical activity (<1 hrs/wk, 1-3 hrs/wk, ≥4 hrs/wk) or post-diagnosis TV viewing (0-2 hrs/d, 3-4 hrs/d, ≥5 hrs/d), and pre-diagnosis physical activity (<1 hrs/wk, 1-3 hrs/wk, ≥4 hrs/wk) or pre-diagnosis TV viewing (0-2 hrs/d, 3-4 hrs/d, ≥5 hrs/d) where appropriate.

Model 3: additionally adjusted for body mass index (18.5-<25.0kg/m², 25.0-<30.0 kg/m², 30.0-<35.kg/m², 35-<65 kg/m²).
