# Supplementary material for: Pre- and post-diagnosis physical activity, television viewing, and mortality among hematologic cancer survivors
Source: PLoS One. 2018 Jan 31;13(1):e0192078. doi: 10.1371/journal.pone.0192078 (PMC5791989; doi:10.1371/journal.pone.0192078)
Supplement: S2 Table — (DOCX) [file pone.0192078.s002.docx]

**S2 Table.** Multivariable-adjusted HRs and 95% CI of all-cause mortality among all individuals diagnosed with hematologic cancer according to physical activity and

TV viewing before and after diagnosis, stratified by age at diagnosis, sex, BMI, and lag time between questionnaire administration and age at diagnosis.

|  | **Pre-diagnosis physical activity** | | | | | | **Pre-diagnosis TV viewing** | | | | | |
| --- | --- | --- | --- | --- | --- | --- | --- | --- | --- | --- | --- | --- |
|  | **<1 hr/wk** | **1 to 3 hrs/wk** | | **≥4 hrs/wk** | **P-interaction** | | **0 to 2 hrs/d** | | **3 to 4 hrs/d** | **≥5 hrs/d** | **P-interaction** | |
| **Age at diagnosis** |  |  | |  |  | |  | |  |  |  | |
| <72 years | 1.00 | 0.85 (0.73-0.98) | | 0.76 (0.67-0.86) | 0.07 | | 1.00 | | 1.07 (0.94-1.21) | 1.21 (1.04-1.41) | 0.08 | |
| ≥72 years | 1.00 | 0.86 (0.72-1.02) | | 0.90 (0.78-1.04) |  | | 1.00 | | 1.16 (1.01-1.33) | 1.07 (0.91-1.26) |  | |
| **Sex** |  |  | |  |  | |  | |  |  |  | |
| Men | 1.00 | 0.87 (0.76-0.99) | | 0.80 (0.72-0.90) | 0.54 | | 1.00 | | 1.06 (0.95-1.19) | 1.10 (0.96-1.26) | 0.29 | |
| Women | 1.00 | 0.82 (0.67-1.003) | | 0.86 (0.72-1.03) |  | | 1.00 | | 1.21 (1.01-1.44) | 1.27 (1.03-1.56) |  | |
| **BMI** |  |  | |  |  | |  | |  |  |  | |
| <25 kg/m² | 1.00 | 0.86 (0.69-1.06) | | 0.80 (0.67-0.96) | 0.87 | | 1.00 | | 1.09 (0.93-1.28) | 1.01 (0.81-1.25) | 0.41 | |
| ≥25 kg/m² | 1.00 | 0.85 (0.75-0.97) | | 0.83 (0.74-0.93) |  | | 1.00 | | 1.11 (0.99-1.25) | 1.19 (1.04-1.36) |  | |
| **Lag time between exposure assessment and diagnosis** |  |  | |  |  | |  | |  |  |  | |
| <7.6 years | 1.00 | 0.87 (0.75-0.995) | | 0.80 (0.71-0.90) | 0.53 | | 1.00 | | 1.06 (0.94-1.19) | 1.11 (0.96-1.28) | 0.42 | |
| ≥7.6 years | 1.00 | 0.82 (0.69-0.98) | | 0.84 (0.73-0.98) |  | | 1.00 | | 1.18 (1.02-1.36) | 1.20 (1.00-1.44) | |  |
|  | **Post-diagnosis physical activity** | | | | | | **Post-diagnosis TV viewing** | | | | | |
|  | **<1 hr/wk** | **1 to <4 hrs/wk** | **≥4 hrs/wk** | | **P-interaction** | | **0 to 2 hrs/d** | | **>2 to 4 hrs/d** | **>4 hrs/d** | | **P-interaction** |
| **Age at diagnosis** |  |  |  | |  | |  | |  |  | |  |
| <69 years | 1.00 | 0.73 (0.54-1.002) | 0.65 (0.47-0.90) | | 0.60 | | 1.00 | | 0.73 (0.53-0.99) | 0.89 (0.64-1.25) | | 0.03 |
| ≥69 years | 1.00 | 0.83 (0.65-1.07) | 0.61 (0.47-0.80) | |  | | 1.00 | | 1.15 (0.88-1.51) | 1.51 (1.15-2.00) | |  |
| **Sex** |  |  |  | |  | |  | |  |  | |  |
| Men | 1.00 | 0.66 (0.52-0.84) | 0.59 (0.47-0.74) | | 0.06 | | 1.00 | | 1.02 (0.81-1.28) | 1.22 (0.95-1.57) | | 0.34 |
| Women | 1.00 | 1.11 (0.78-1.58) | 0.70 (0.45-1.08) | |  | | 1.00 | | 0.75 (0.50-1.13) | 1.21 (0.83-1.78) | |  |
| **BMI** |  |  |  | |  | |  | |  |  | |  |
| <25 kg/m² | 1.00 | 0.78 (0.54-1.13) | 0.54 (0.36-0.79) | | 0.52 | | 1.00 | | 1.01 (0.70-1.44) | 1.84 (1.22-2.78) | | 0.22 |
| ≥25 kg/m² | 1.00 | 0.74 (0.59-0.94) | 0.64 (0.50-0.82) | |  | | 1.00 | | 0.91 (0.71-1.16) | 1.04 (0.81-1.34) | |  |
| **Lag time between diagnosis and exposure assessment** |  |  |  | |  |  | |  | |  | |  |
| <3.4 years | 1.00 | 0.75 (0.57-0.98) | 0.62 (0.47-0.82) | | 0.94 | 1.00 | | 1.04 (0.79-1.37) | | 1.13 (0.83-1.53) | | 0.23 |
| ≥3.4 years | 1.00 | 0.75 (0.57-1.001) | 0.57 (0.42-0.77) | |  | 1.00 | | 0.84 (0.62-1.13) | | 1.28 (0.95-1.73) | |  |

HR=hazard ratio, CI=confidence interval, TV= television, BMI=body mass index

Multivariable models are adjusted for age at exposure assessment (continuous), age at cancer diagnosis (continuous), education (less than 12 yrs, 12 yrs, vocational training or some college education, college graduate/postgraduate, unknown), race (non-Hispanic White, non-Hispanic Black, other, unknown), smoking (never smoker, former smoker with 20 cigarettes per day or less, former smoker with more than 20 cigarettes per day, current smoker with 20 cigarettes per day or less, current smoker with more than 20 cigarettes per day, missing), alcohol consumption (0, >0 to 14.9, ≥15g/d), hematologic cancer subtype (NHL, HL, myeloma, leukemia) and stage in NHL survivors (localized/regional/in situ, systemic disease, unknown/not abstracted/missing), chemotherapy (yes, no, unknown/missing), and physical activity or TV viewing where appropriate.
