# Supplementary material for: Pre- and post-diagnosis physical activity, television viewing, and mortality among hematologic cancer survivors
Source: PLoS One. 2018 Jan 31;13(1):e0192078. doi: 10.1371/journal.pone.0192078 (PMC5791989; doi:10.1371/journal.pone.0192078)
Supplement: S3 Table — (DOCX) [file pone.0192078.s003.docx]

**S3 Table.** Multivariable-adjusted HRs and 95% CI for the relation of post-diagnosis physical activity or TV viewing to all-cause mortality among all individuals diagnosed with hematologic cancer according to pre-diagnosis physical activity or TV viewing.

|  | **Post-diagnosis physical activity** | | | |
| --- | --- | --- | --- | --- |
|  | **<1 hr/wk** | **1 to <4 hrs/wk** | **≥4 hrs/wk** | **P-interaction** |
| **Pre-diagnosis physical activity** |  |  |  |  |
| **<1 hr/wk** | 1.00 | 0.98 (0.62-1.54) | 0.59 (0.32-1.08) | 0.53 |
| **1 to 3 hrs/wk** | 1.00 | 0.69 (0.44-1.08) | 0.46 (0.27-0.80) |  |
| **≥4 hrs/wk** | 1.00 | 1.02 (0.70-1.49) | 0.79 (0.54-1.15) |  |
|  | **Post-diagnosis TV viewing** | | | |
|  | **0 to 2 hrs/d** | **>2 to 4 hrs/d** | **>4 hrs/d** | **P-interaction** |
| **Pre-diagnosis TV viewing** |  |  |  |  |
| **0 to 2 hrs/d** | 1.00 | 1.03 (0.67-1.57) | 0.72 (0.39-1.31) | 0.20 |
| **3 to 4 hrs/d** | 1.00 | 0.87 (0.61-1.24) | 1.27 (0.84-1.91) |  |
| **≥5 hrs/d** | 1.00 | 0.87 (0.29-2.62) | 2.96 (1.11-7.91) |  |

HR=hazard ratio, CI=confidence interval, TV= television

Multivariable models are adjusted for age at exposure assessment (continuous), age at cancer diagnosis (continuous), education (less than 12 yrs, 12 yrs, vocational training or some college education, college graduate/postgraduate, unknown), race (non-Hispanic White, non-Hispanic Black, other, unknown), smoking (never smoker, former smoker with 20 cigarettes per day or less, former smoker with more than 20 cigarettes per day, current smoker with 20 cigarettes per day or less, current smoker with more than 20 cigarettes per day, missing), alcohol consumption (0, >0 to 14.9, ≥15g/d), hematologic cancer subtype (NHL, HL, myeloma, leukemia) and stage in NHL survivors (localized/regional/in situ, systemic disease, unknown/not abstracted/missing), chemotherapy (yes, no, unknown/missing), and physical activity or TV viewing where appropriate.
