# Supplementary material for: Pre- and post-diagnosis physical activity, television viewing, and mortality among hematologic cancer survivors
Source: PLoS One. 2018 Jan 31;13(1):e0192078. doi: 10.1371/journal.pone.0192078 (PMC5791989; doi:10.1371/journal.pone.0192078)
Supplement: S5 Table — (DOCX) [file pone.0192078.s005.docx]

**S5 Table.** Multivariable-adjusted HRs and 95% CI of all-cause mortality among individuals diagnosed with hematologic cancer according to TV viewing before and after diagnosis, additionally adjusted for self-reported health status.

|  | **Pre-diagnosis TV viewing** | | | | **Post-diagnosis TV viewing** | | | | |
| --- | --- | --- | --- | --- | --- | --- | --- | --- | --- |
|  | **0 to 2 hrs/d** | **3 to 4 hrs/d** | **≥5 hrs/d** | ***p*-trend** | **0 to 2 hrs/d** | **>2 to 4 hrs/d** | **>4 hrs/d** | | ***p*-trend** |
| **All hematologic cancer survivors** |  |  |  |  |  |  | |  |  |
| Deaths | 774 | 1239 | 593 |  | 201 | 199 | | 177 |  |
| Multivariable-adjusted HR (95% CI) | 1.00 | 1.09 (0.99-1.19) | 1.10 (0.98-1.23) | 0.07 | 1.00 | 0.91 (0.74-1.10) | | 1.05 (0.85-1.30) | 0.81 |
| Multivariable- and BMI-adjusted HR (95% CI) | 1.00 | 1.08 (0.98-1.18) | 1.08 (0.96-1.20) | 0.16 | 1.00 | 0.91 (0.74-1.11) | | 1.05 (0.85-1.31) | 0.80 |
| **Non-Hodgkin lymphoma survivors** |  |  |  |  |  |  | |  |  |
| Deaths | 337 | 524 | 247 |  | 100 | 96 | | 76 |  |
| Multivariable-adjusted HR (95% CI) | 1.00 | 1.16 (1.01-1.34) | 1.20 (1.01-1.43) | 0.03 | 1.00 | 0.99 (0.74-1.32) | | 0.99 (0.72-1.36) | 0.96 |
| Multivariable- and BMI-adjusted HR (95% CI) | 1.00 | 1.15 (0.999-1.33) | 1.19 (0.99-1.41) | 0.04 | 1.00 | 0.99 (0.74-1.32) | | 0.99 (0.72-1.37) | 0.94 |
| **Myeloma survivors** |  |  |  |  |  |  | |  |  |
| Deaths | 173 | 244 | 119 |  | 41 | 36 | | 33 |  |
| Multivariable-adjusted HR (95% CI) | 1.00 | 0.85 (0.69-1.04) | 0.78 (0.60-1.01) | 0.046 | 1.00 | 0.86 (0.51-1.44) | | 0.90 (0.53- 1.55) | 0.69 |
| Multivariable- and BMI-adjusted HR (95% CI) | 1.00 | 0.84 (0.69-1.03) | 0.76 (0.58-0.98) | 0.03 | 1.00 | 0.86 (0.51-1.44) | | 0.87 (0.50-1.51) | 0.59 |
| **Leukemia survivors** |  |  |  |  |  |  | |  |  |
| Deaths | 253 | 446 | 211 |  | 55 | 61 | | 63 |  |
| Multivariable-adjusted HR (95% CI) | 1.00 | 1.14 (0.97-1.33) | 1.16 (0.95-1.40) | 0.11 | 1.00 | 0.78 (0.53-1.14) | | 1.14 (0.76-1.71) | 0.63 |
| Multivariable- and BMI-adjusted HR (95% CI) | 1.00 | 1.13 (0.96-1.33) | 1.13 (0.93-1.38) | 0.17 | 1.00 | 0.78 (0.53-1.15) | | 1.18 (0.78-1.78) | 0.58 |
| **Acute leukemia survivors*** |  |  |  |  |  |  | |  |  |
| Deaths | 135 | 224 | 111 |  |  |  | |  |  |
| Multivariable-adjusted HR (95% CI) | 1.00 | 1.31 (1.05-1.64) | 1.42 (1.09-1.85) | 0.01 | 1.00 | - | | - | - |
| Multivariable- and BMI-adjusted HR (95% CI) | 1.00 | 1.30 (1.03-1.63) | 1.37 (1.05-1.80) | 0.01 | 1.00 | - | | - | - |
| **Chronic leukemia survivors** |  |  |  |  |  |  | |  |  |
| Deaths | 104 | 190 | 82 |  | 42 | 49 | | 54 |  |
| Multivariable-adjusted HR (95% CI) | 1.00 | 1.14 (0.88-1.46) | 0.95 (0.70-1.29) | 0.89 | 1.00 | 0.75 (0.48-1.18) | | 1.23 (0.78-1.93) | 0.42 |
| Multivariable- and BMI-adjusted HR (95% CI) | 1.00 | 1.11 (0.86-1.44) | 0.92 (0.67-1.27) | 0.74 | 1.00 | 0.78 (0.50-1.21) | | 1.33 (0.82-2.13) | 0.31 |

HR=hazard ratio, CI=confidence interval, TV= television, BMI=body mass index

Multivariable models adjusted for age at exposure assessment (continuous), age at cancer diagnosis (continuous), sex, education (less than 12 yrs, 12 yrs, vocational training or some college education, college graduate/postgraduate, unknown), race (non-Hispanic White, non-Hispanic Black, other, unknown), smoking (never smoker, former smoker with 20 cigarettes per day or less, former smoker with more than 20 cigarettes per day, current smoker with 20 cigarettes per day or less, current smoker with more than 20 cigarettes per day, missing), alcohol consumption (0, >0 to 14.9, ≥15g/d), chemotherapy (yes, no, unknown/missing), hematologic cancer subtype (NHL, HL, myeloma, leukemia) and stage in NHL survivors (localized/regional/in situ, systemic disease, unknown/not abstracted/missing), physical activity, and self-reported health (poor/fair, good, very good/excellent). *Data were not evaluated for post-diagnosis TV viewing due to low sample size.
