# Supplementary material for: Pre- and post-diagnosis physical activity, television viewing, and mortality among hematologic cancer survivors
Source: PLoS One. 2018 Jan 31;13(1):e0192078. doi: 10.1371/journal.pone.0192078 (PMC5791989; doi:10.1371/journal.pone.0192078)
Supplement: S7 Table — (DOCX) [file pone.0192078.s007.docx]

**S7 Table.** Multivariable-adjusted HRs and 95% CI of mortality according to TV viewing before and after diagnosis among hematologic cancer survivors after exclusion of participants who answered the questionnaire within 12 months of death or participants diagnosed with hematologic cancer within one year of exposure assessment.

|  | **Pre-diagnosis TV viewing** | | | | **Post-diagnosis TV viewing** | | | |
| --- | --- | --- | --- | --- | --- | --- | --- | --- |
|  | **0 to 2 hrs/d** | **3 to 4 hrs/d** | **≥5 hrs/d** | ***p*-trend** | **0 to 2 hrs/d** | **>2 to 4 hrs/d** | **>4 hrs/d** | ***p*-trend** |
| **All hematologic cancer survivors** |  |  |  |  |  |  |  |  |
| **All-cause mortality** |  |  |  |  |  |  |  |  |
| Deaths^a^ | 765 | 1218 | 584 |  | 172 | 174 | 145 |  |
| Multivariable-adjusted HR (95% CI)^a^ | 1.00 | 1.10 (1.01-1.21) | 1.14 (1.02-1.28) | 0.01 | 1.00 | 0.95 (0.77-1.18) | 1.16 (0.92-1.46) | 0.29 |
| Multivariable + BMI-adjusted HR (95%CI)^a^ | 1.00 | 1.09 (0.99-1.19) | 1.11 (0.99-1.24) | 0.06 | 1.00 | 0.96 (0.77-1.19) | 1.17 (0.93-1.47) | 0.27 |
| Deaths^b^ | 710 | 1159 | 540 |  | 155 | 159 | 146 |  |
| Multivariable-adjusted HR (95% CI)^b^ | 1.00 | 1.13 (1.03-1.25) | 1.15 (1.02-1.29) | 0.01 | 1.00 | 0.93 (0.74-1.16) | 1.22 (0.96-1.54) | 0.16 |
| Multivariable + BMI-adjusted HR (95% CI)^b^ |  | 1.12 (1.01-1.23) | 1.11 (0.99-1.25) | 0.04 | 1.00 | 0.93 (0.74-1.17) | 1.22 (0.96-1.55) | 0.16 |
| **Hematologic cancer mortality** |  |  |  |  |  |  |  |  |
| Deaths^a^ | 546 | 831 | 384 |  | 92 | 94 | 69 |  |
| Multivariable-adjusted HR (95% CI)^a^ | 1.00 | 1.07 (0.96-1.20) | 1.10 (0.96-1.26) | 0.15 | 1.00 | 0.99 (0.74-1.33) | 1.09 (0.79-1.51) | 0.65 |
| Multivariable + BMI-adjusted HR (95%CI)^a^ | 1.00 | 1.06 (0.95-1.18) | 1.06 (0.93-1.22) | 0.33 | 1.00 | 0.998 (0.74-1.34) | 1.12 (0.80-1.56) | 0.56 |
| Deaths^b^ | 507 | 788 | 360 |  | 81 | 87 | 72 |  |
| Multivariable-adjusted HR (95% CI)^b^ | 1.00 | 1.10 (0.98-1.23) | 1.12 (0.97-1.29) | 0.09 | 1.00 | 1.00 (0.73-1.36) | 1.22 (0.88-1.70) | 0.27 |
| Multivariable + BMI-adjusted HR (95% CI)^b^ | 1.00 | 1.09 (0.97-1.22) | 1.09 (0.94-1.25) | 0.20 | 1.00 | 1.003 (0.74-1.37) | 1.25 (0.89-1.75) | 0.24 |
| **Non-Hodgkin lymphoma survivors** |  |  |  |  |  |  |  |  |
| **All-cause mortality** |  |  |  |  |  |  |  |  |
| Deaths^a^ | 334 | 516 | 245 |  | 85 | 85 | 65 |  |
| Multivariable-adjusted HR (95% CI)^a^ | 1.00 | 1.17 (1.02-1.35) | 1.26 (1.06-1.50) | 0.01 | 1.00 | 1.08 (0.80-1.47) | 1.18 (0.84-1.65) | 0.35 |
| Multivariable + BMI-adjusted HR (95%CI)^a^ |  | 1.16 (1.01-1.34) | 1.23 (1.03-1.47) | 0.01 | 1.00 | 1.08 (0.79-1.47) | 1.17 (0.83-1.65) | 0.37 |
| Deaths^b^ | 308 | 493 | 221 |  | 76 | 84 | 62 |  |
| Multivariable-adjusted HR (95% CI)^b^ | 1.00 | 1.22 (1.05-1.41) | 1.25 (1.04-1.50) | 0.01 | 1.00 | 1.12 (0.82-1.54) | 1.15 (0.81-1.64) | 0.39 |
| Multivariable + BMI-adjusted HR (95% CI)^b^ | 1.00 | 1.20 (1.04-1.39) | 1.22 (1.02-1.47) | 0.02 | 1.00 | 1.13 ( 0.82-1.56) | 1.15 (0.80-1.65) | 0.41 |
| **Myeloma survivors** |  |  |  |  |  |  |  |  |
| **All-cause mortality** |  |  |  |  |  |  |  |  |
| Deaths^a^ | 173 | 241 | 119 |  | 38 | 35 | 27 |  |
| Multivariable-adjusted HR (95% CI)^a^ | 1.00 | 0.85 (0.69-1.04) | 0.79 (0.61-1.02) | 0.06 | 1.00 | 0.87 (0.51-1.49) | 0.84 (0.47-1.49) | 0.54 |
| Multivariable + BMI-adjusted HR (95%CI)^a^ |  | 0.84 (0.68-1.03) | 0.76 (0.59-0.98) | 0.03 |  | 0.88 (0.52-1.50) | 0.83 (0.46-1.48) | 0.52 |
| Deaths^b^ | 164 | 230 | 111 |  | 32 | 25 | 25 |  |
| Multivariable-adjusted HR (95% CI)^b^ | 1.00 | 0.84 (0.68-1.04) | 0.78 (0.60-1.02) | 0.05 | 1.00 | 0.68 (0.37-1.26) | 0.84 (0.44-1.60) | 0.52 |
| Multivariable + BMI-adjusted HR (95% CI)^b^ | 1.00 | 0.84 (0.68-1.03) | 0.75 (0.58-0.98) | 0.03 | 1.00 | 0.71 (0.38-1.33) | 0.82 (0.42-1.62) | 0.48 |
| **Leukemia survivors** |  |  |  |  |  |  |  |  |
| **All-cause mortality** |  |  |  |  |  |  |  |  |
| Deaths^a^ | 247 | 436 | 204 |  | 45 | 48 | 50 |  |
| Multivariable-adjusted HR (95% CI)^a^ | 1.00 | 1.16 (0.99-1.37) | 1.18 (0.97-1.43) | 0.07 | 1.00 | 0.86 (0.57-1.31) | 1.49 (0.96-2.30) | 0.12 |
| Multivariable + BMI-adjusted HR (95%CI)^a^ | 1.00 | 1.15 (0.98-1.35) | 1.14 (0.94-1.39) | 0.15 | 1.00 | 0.88 (0.57-1.34) | 1.59 (1.01-2.49) | 0.09 |
| Deaths^b^ | 228 | 412 | 196 |  | 42 | 44 | 54 |  |
| Multivariable-adjusted HR (95% CI)^b^ |  | 1.22 (1.03-1.44) | 1.23 (1.003-1.50) | 0.03 | 1.00 | 0.72 (0.47-1.12) | 1.58 (1.03-2.42) | 0.07 |
| Multivariable + BMI-adjusted HR (95% CI)^b^ |  | 1.20 (1.02-1.43) | 1.19 (0.97-1.46) | 0.06 | 1.00 | 0.72 (0.46-1.12) | 1.68 (1.08-2.61) | 0.06 |
| **Acute leukemia survivors*** |  |  |  |  |  |  |  |  |
| **All-cause mortality** |  |  |  |  |  |  |  |  |
| Deaths^a^ | 130 | 216 | 105 |  |  |  |  |  |
| Multivariable-adjusted HR (95% CI)^a^ | 1.00 | 1.27 (1.01-1.59) | 1.45 (1.11-1.90) | 0.01 | 1.00 | - | - | - |
| Multivariable + BMI-adjusted HR (95%CI)^a^ |  | 1.24 (0.99-1.56) | 1.38 (1.05-1.81) | 0.02 |  |  |  |  |
| Deaths^b^ | 126 | 210 | 103 |  |  |  |  |  |
| Multivariable-adjusted HR (95% CI)^b^ | 1.00 | 1.31 (1.04-1.65) | 1.46 (1.11-1.92) | 0.004 | 1.00 | - | - | - |
| Multivariable + BMI-adjusted HR (95% CI)^b^ |  | 1.28 (1.02-1.62) | 1.39 (1.06-1.84) | 0.01 |  | - | - | - |
| **Chronic leukemia survivors** |  |  |  |  |  |  |  |  |
| **All-cause mortality** |  |  |  |  |  |  |  |  |
| Deaths^a^ | 103 | 189 | 81 |  | 36 | 39 | 44 |  |
| Multivariable-adjusted HR (95% CI)^a^ | 1.00 | 1.17 (0.91-1.50) | 0.98 (0.72-1.33) | 0.90 | 1.00 | 0.81 (0.50-1.30) | 1.58 (0.98-2.55) | 0.09 |
| Multivariable + BMI-adjusted HR (95%CI)^a^ |  | 1.14 (0.88-1.47) | 0.94 (0.69-1.29) | 0.86 | 1.00 | 0.83 (0.52-1.35) | 1.69 (1.02-2.80) | 0.07 |
| Deaths^b^ | 92 | 171 | 76 |  | 33 | 37 | 46 |  |
| Multivariable-adjusted HR (95% CI)^b^ | 1.00 | 1.23 (0.95-1.60) | 1.06 (0.77-1.46) | 0.55 | 1.00 | 0.76 (0.46-1.23) | 1.63 (1.02-2.62) | 0.07 |
| Multivariable + BMI-adjusted HR (95% CI)^b^ |  | 1.21 (0.93-1.58) | 1.03 (0.74-1.43) | 0.70 | 1.00 | 0.76 (0.47-1.25) | 1.74 (1.05-2.88) | 0.06 |

HR=hazard ratio, CI=confidence interval, BMI=body mass index

Multivariable models adjusted for age at exposure assessment (continuous), age at cancer diagnosis (continuous), sex, education (less than 12 yrs, 12 yrs, vocational training or some college education, college graduate/postgraduate, unknown), race (non-Hispanic White, non-Hispanic Black, other, unknown), smoking (never smoker, former smoker with 20 cigarettes per day or less, former smoker with more than 20 cigarettes per day, current smoker with 20 cigarettes per day or less, current smoker with more than 20 cigarettes per day, missing), alcohol consumption (0, >0 to 14.9, ≥15g/d), chemotherapy (yes, no, unknown/missing), hematologic cancer subtype (NHL, HL, myeloma, leukemia) and stage in NHL survivors (localized/regional/in situ, systemic disease, unknown/not abstracted/missing), and physical activity. *Data were not evaluated for post-diagnosis physical activity due to low sample size. a) Participants who answered the questionnaire within 12 months of death excluded. b) Participants diagnosed with hematologic cancer within one year of exposure assessment excluded.
